# A welding phenomenon of dissimilar nanoparticles in dispersion

**DOI:** 10.1038/s41467-018-08206-6

**Published:** 2019-01-15

**Authors:** Zhiqi Huang, Zhi-Jian Zhao, Qian Zhang, Lili Han, Xiumei Jiang, Chao Li, Maria T. Perez Cardenas, Peng Huang, Jun-Jie Yin, Jun Luo, Jinlong Gong, Zhihong Nie

**Affiliations:** 1 Key Laboratory for Green Chemical Technology of Ministry of Education, School of Chemical Engineering and Technology, Collaborative Innovation Center of Chemical Science and Engineering, Tianjin University, 300072 Tianjin, China; 2 Department of Chemistry and Biochemistry, University of Maryland, College Park, MD 20742 USA; 3 Center for Electron Microscopy TUT-FEI Joint Laboratory, Institute for New Energy Materials & Low-Carbon Technologies; School of Materials Science and Engineering, Tianjin University of Technology, 300384 Tianjin, China; 4 Division of Analytical Chemistry, Office of Regulatory Science, Center for Food Safety and Applied Nutrition, U.S. Food and Drug Administration, College Park, MD 20740 USA; 5 Guangdong Key Laboratory for Biomedical Measurements and Ultrasound Imaging, Department of Biomedical Engineering, School of Medicine, Shenzhen University, 518060 Shenzhen, China; 6 State Key Laboratory of Molecular Engineering of Polymers, Department of Macromolecular Science, Fudan University, Shanghai, 200438 P.R., China

## Abstract

The oriented attachment of small nanoparticles (NPs) is recognized as an important mechanism involved in the growth of inorganic nanocrystals. However, non-oriented attachment of dissimilar NPs has been rarely observed in dispersion. This communication reports a welding phenomenon occurred directly between as-synthesized dispersions of single-component Au and chalcogenide NPs, which leads to the formation of asymmetric Au-chalcogenide hybrid NPs (HNPs). The welding of dissimilar NPs in dispersion is mainly driven by the ligand desorption-induced conformal contact between NPs and the diffusion of Au into chalcogenide NPs. The welding process can occur between NPs with distinct shapes or different capping agents or in different solvent media. A two-step assembly-welding mechanism is proposed for this process, based on our in situ electron spin resonance measurements and ab initio molecular dynamics simulation. The understanding of NP welding in dispersion may lead to the development of unconventional synthetic tools for the fabrication of hybrid nanostructures with diverse applications.

## Abstract (web summary)

Solution-phase welding of nanoparticles to form larger structures typically requires that the particles are the same type, limiting the diversity of possible products. Here, the authors report a welding process between gold and chalcogenide nanoparticles in dispersion that leads to asymmetric hybrid nanoparticles with two distinct domains.

## Introduction

Colloidal inorganic hybrid nanoparticles (NPs) (HNPs) are multicomponent colloids with dissimilar inorganic components integrated within one colloidal particle. Compared with single-component NPs, HNPs not only combine chemical and physical properties of each component, but also may exhibit new synergistic properties that are not attainable by any of their individual constituent components^1–4^. As a result, HNPs have outperformed single-component NPs in a variety of applications ranging from energy^5–7^ to biomedicine^8^. Recently, enormous progress has been made in the wet-chemical synthesis of HNPs with different material combinations^9–11^. The dominating mechanism involved in such synthesis is the so-called seed-mediated growth in which a secondary component monomer nucleates on the surface of a primary component seed^12,13^. During the synthesis, a delicate control over the growth kinetics and interfacial energy between multiple components is typically required to obtain HNPs with desired topology and morphology^14–16^. This, however, represents a synthetic challenge for achieving reproducible and scalable production of HNPs. Therefore, there is an urgent need to develop novel synthetic approaches beyond seeded-mediated growth for the synthesis of high-quality HNPs.

The oriented attachment of small NPs (e.g., semiconductors, metals) is recognized as an important mechanism involved in the growth of inorganic nanocrystals^17^. Recently, a broad range of complex nanostructures such as one-dimensional nanowires (NWs)^18,19^ and two-dimensional nanosheets^20,21^ has been synthesized via the oriented attachment. The welding of NPs to form larger nanostructures in this process is largely driven by the instability of NPs and the elimination of common crystal facets of neighboring nanocrystals. This phenomenon, however, has been usually limited to the welding of NPs of the same kind, due to the general requirement on lattice matching. In contrast, the non-oriented attachment mechanism not only occurs between the same type of NPs^22,23,^ but also allows for the welding of dissimilar NPs, thus facilitating the synthesis of HNPs with diverse compositions. For example, the welding of Au and Ag NPs was observed upon contact in vacuum at room temperature^24^. However, the non-oriented attachment between dissimilar NPs in dispersion has rarely been reported. One example involves the random aggregation and uncontrollable welding of CuSe and In_2_S_3_ NPs in dispersion to produce single-component CuInS_*x*_Se_1−*x*_ NPs with irregular shapes^25^, rather than multicomponent hybrid nanostructures with distinct domains. To date, there has been no report on the successful welding of dissimilar NPs in dispersions to synthesize multicomponent HNPs.

Here, we describe a welding phenomenon occurred directly between as-synthesized single-component Au and chalcogenide (e.g., Ag_2_S, Ag_2_Se) NPs in dispersion, which leads to the formation of asymmetric Au–chalcogenide HNPs. This welding process does not require lattice matching between NPs and exhibited obvious atom diffusion, which are characteristics of non-oriented attachment mechanism. The diffusion of Au into Ag_2_S domains is observed in the derived HNPs and is believed to be the driven force of the welding process^26^. This mechanism is further confirmed by molecular dynamic simulation, which shows the penetration of Au atoms into Ag_2_S lattice at the Au–Ag_2_S interface. The welding process is found to be largely dependent on the ligand desorption that brings dissimilar NPs into close proximity. We monitor the welding process in situ by quantifying the photoreduction of a nitroxyl radical 2,2,6,6-tetramethylpiperidine-1-oxyl (TEMPO) using electron spin resonance (ESR) spectroscopy. This wedding phenomenon is also observed between NPs with distinct shapes. When Au nanorods (NRs) and Au NWs are used, their welding with spherical Ag_2_S NPs produce matchstick-like or tree burl-like HNPs. Through the current study, we have demonstrated the merging of dissimilar NPs in dispersions to form multicomponent HNPs with defined shapes and morphologies by controlling the ligands on the surface of initial NPs. We have also identified a new two-step assembly-diffusion mechanism for the production of HNPs, which distinguishes our study from others. This work represents an important step toward bottom-up synthesis of complex multicomponent HNPs with desired morphologies and properties via controlled NP welding.

## Results

### Welding of spherical Au and Ag_2_S NPs

**Fig. 1:**
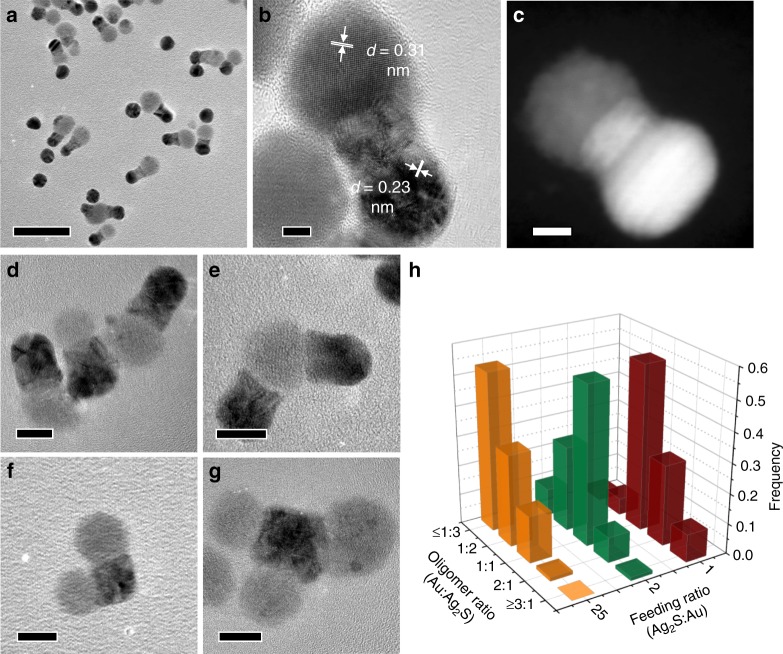
Formation of Au–Ag_2_S oligomer-like HNPs through welding of spherical Au and Ag_2_S NPs. **a** TEM image of welded Au–Ag_2_S HNPs, **b** HRTEM, and **c** HAADF-STEM images of individual Au–Ag_2_S dimer formed by welding Au and Ag_2_S NPs in dispersion. **d**–**g** TEM images of Au–Ag_2_S HNPs with different Au:Ag_2_S domain ratios: **d** 1:1, **e** 2:1, **f** 1:2, and **g** 1:3. **h** Statistical histogram showing the Au:Ag_2_S domain ratio of derived Au–Ag_2_S HNPs at different feeding ratios of Ag_2_S:Au NPs. Scale bars are 50 nm in **a**, 5 nm in **b**, **c**, and 10 nm in **d**–**g**

Spherical Au NPs of 14.0 ± 1.3 nm in diameter were prepared by a citrate-reduction method^27^ (Supplementary Fig. 1) and surface-modified with thiol-terminated poly (ethylene glycol) (PEG-SH, *M*_w_ = 5 k) with controlled density (Au-1 to Au-4, Supplementary Table 1). Spherical Ag_2_S NPs of 16.0 ± 2.9 nm in diameter were synthesized using a bovine serum albumin (BSA)-directed growth method^28^ (Supplementary Fig. 2) and were washed several times to remove excess BSA before use. Au NPs with low density of PEG and Ag_2_S NPs were then mixed in a predetermined ratio and incubated at ambient condition for 24 h to allow for the welding. Figure 1a shows the representative transmission electron microscopy (TEM) image of the derived oligomer-like Au–Ag_2_S HNPs. All the Ag_2_S NPs are attached to Au NPs to form HNPs in which Au and Ag_2_S domains can be readily distinguished (dark: Au; gray: Ag_2_S). The 0.23 and 0.31 nm d-spacing can be assigned to the (111) facet of Au and (121) facet of Ag_2_S, respectively (Fig. 1b). High-resolution TEM (HRTEM) image (Fig. 1b) and high-angle annular dark-field scanning TEM (HAADF-STEM) image (Fig. 1c) of an individual welded Au–Ag_2_S HNP clearly show that the Au and Ag_2_S domains are fused together rather than physically attached or overlapped due to drying effect. In between the Au and Ag_2_S domain, there is a diffusion region where Au gradually diffuses into Ag_2_S domain, as indicated by the spread of dark Au domain into gray Ag_2_S domain. The successful welding of Au and Ag_2_S NPs in dispersion was also confirmed by the red-shift of the plasmonic peak of Au NPs from 510 to 525 nm in the UV/Vis spectroscopy analysis (Supplementary Fig. 3). The spectra of Au and Ag_2_S mixture (0, 2, and 24 h) showed strong absorption below 500 nm, compared with that of pure Au NP dispersion. This is due to the summation of the absorption of Au and Ag_2_S NPs. Moreover, upon mixing, the electrostatic interaction between oppositely charged Au and Ag_2_S NPs immediately induced the assembly of dissimilar NPs and brought the Ag_2_S NPs to the close proximity of Au NPs. The red-shift of the absorption spectra can thus be ascribed to the increase of local refractive index around Au NPs after Ag_2_S NP attachment^29^.

We note that the number of Au and Ag_2_S domains within the welded HNPs does not exactly follow the feeding ratio of building blocks. As an example, at 1:1 ratio (volume of NP dispersion), the yield of dimers composed of one Au and one Ag_2_S domain (Fig. 1d) is ~55%. HNPs with other morphologies were also present in the product: 27% of trimers composed of two Au and one Ag_2_S domains (Fig. 1e), 9% of tetramers composed of three Au and one Ag_2_S domains, 6% of trimers composed of one Au and two Ag_2_S domains (Fig. 1f), and 3% of tetramers composed of one Au and three Ag_2_S domains (Fig. 1g). We attribute this relatively wide distribution of HNPs to the probability of collision and contact between disparate NPs in dispersion. After the formation of dimers with one Au and one Ag_2_S domains, they may further interact with either Au or Ag_2_S NPs in the system to form HNPs with more than two domains. Nevertheless, the product distribution could be roughly tuned by varying the feeding ratio of dissimilar NPs. As shown in Fig. 1h, increasing the feeding ratio of Ag_2_S:Au NPs led to the formation of HNPs with a larger number of Ag_2_S domains in average within individual HNPs (Supplementary Fig. 4).

The grafting of PEG on Au NP surface is essential for stabilizing the colloidal particles during their welding in dispersion. More importantly, the grafting density of PEG determines the onset and rate of welding process. Without PEG modification (Au-0), the negatively charged Au NPs (zeta potential of −43 mV) strongly attract positively charged Ag_2_S NPs (zeta potential of +32 mV), thus leading to irreversible aggregation and precipitation of these NPs in dispersion^30^ (Supplementary Fig. 5a). The presence of PEG weakened the surface charges on the NPs (Au-1, 2): the absolute values of zeta potential of NPs decreased significantly from about −45 to −15 mV with increasing PEG coverage on Au NPs from 0 to ~0.7 chains nm^−2^ (Supplementary Fig. 6). This can be explained by the replacement of negatively charged citrate by neutral PEG and the burying of charged citrate under the PEG layer. It is worth noting that the welding only occurs for Au NPs with a moderate polymer coverage (~0.05–0.11 chain nm^−2^) (Supplementary Table 1 and Supplementary Fig. 5b). A high density of PEG (>~0.36 chain nm^−2^) hinders the welding process (Supplementary Table 1 and Supplementary Fig. 5c), as covalently bonded PEG chains are stable on Au surface and barely drop off from Au surfaces^31,32^.

### Mechanism investigation of the welding process

**Fig. 2:**
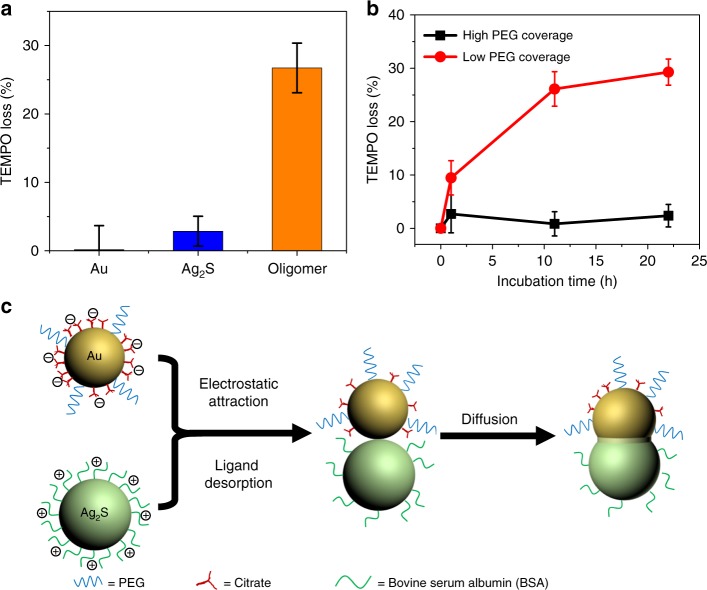
Mechanism investigation of the welding process using ESR. **a** A comparison of TEMPO loss in the dispersion of Au NPs, Ag_2_S NPs, and Au–Ag_2_S HNPs, after the irradiation with visible light for 15 min. **b** TEMPO loss for incubated Ag_2_S and Au NPs with different polymer coverage as a function of incubation time. Each test is repeated three times. Standard deviations are represented by error bars. **c** Schematic illustration of the hypothesized welding mechanism

In contrast to pristine Au and Ag_2_S NPs, the presence of a Schottky barrier at the Au–Ag_2_S interface promotes the hot electron-hole separation and prolongs the lifetime of charge carriers in photo-irradiated Au–Ag_2_S HNPs. The photo-generated hot electrons can be detected by ESR spectroscopy using nitroxyl radical TEMPO as a probe^33,34^. We compared the loss of TEMPO (i.e., reduction of TEMPO by hot electrons) in the dispersion of Au NPs, Ag_2_S NPs, and Au–Ag_2_S HNPs upon irradiation with visible light. As shown in Fig. 2a, the loss of TEMPO was significant in welded Au–Ag_2_S HNPs. In contrast, the loss of TEMPO was not obvious in the dispersion of either pristine Au or Ag_2_S NPs, regardless of the ligand coverage (Supplementary Fig. 7). We, therefore, used ESR to monitor the welding of Au and Ag_2_S NPs in situ in dispersion and rule out the possibility of electron beam-accelerated welding of NPs^35^. After different incubation time, an equivalent of the mixture of Au and Ag_2_S NPs was mixed with the same amount of TEMPO and irradiated with visible light. As shown in Fig. 2b, for Au NPs with low PEG coverage, the loss of TEMPO increased with increasing incubation time, indicating the continuous welding of NPs. The welding proceeded gradually and mostly completed in ~12 h of incubation, as indicated by a plateau in Fig. 2b. In contrast, no significant change in the loss of TEMPO was observed in the system containing Au NPs with high PEG coverage. This further confirmed that the PEG coverage on NPs plays a crucial role in the occurrence of NP welding.

Taken together, these results suggest an assembly-diffusion mechanism of welding (Fig. 2c). Upon mixing the dispersion of Au and Ag_2_S NPs, the weak electrostatic attraction between dissimilar NPs and repulsion between the same types of NPs (i.e., between Au and Au NPs or between Ag_2_S and Ag_2_S NPs) facilitate the hetero-clustering of NPs. During the incubation, residual citrates weakly bonded on Au NPs gradually desorb to induce conformal contact and atomic diffusion of Au and Ag_2_S NPs^18^. The placement of Au and Ag_2_S NPs in close proximity initiates atom diffusion between Au and Ag_2_S domain and eventually the welding of NPs to produce HNPs^35–37^. The diffusion was supported by the gradual spreading of Au signal from its original spherical shape toward Ag_2_S domain with gradient signal intensity in energy-dispersive X-ray spectroscopy (EDS) mapping of the Au–Ag_2_S HNP (Supplementary Fig. 8a–d). Unfortunately, the overlap between Au and S signal makes it impossible to quantify the distribution of S within the HNPs (Supplementary Fig. 9)^38^. Moreover, X-ray powder diffraction (XRD) results suggested the presence of trace Ag_3_AuS_2_ compounds in the welded HNPs, which further confirms the diffusion of Au (Supplementary Fig. 10). The corresponding peaks of Ag_3_AuS_2_ were distinctive from those of Au and Ag_2_S, although they were much weaker than those of Au and Ag_2_S due to the very small welding region in individual Au–Ag_2_S HNPs.

**Fig. 3:**
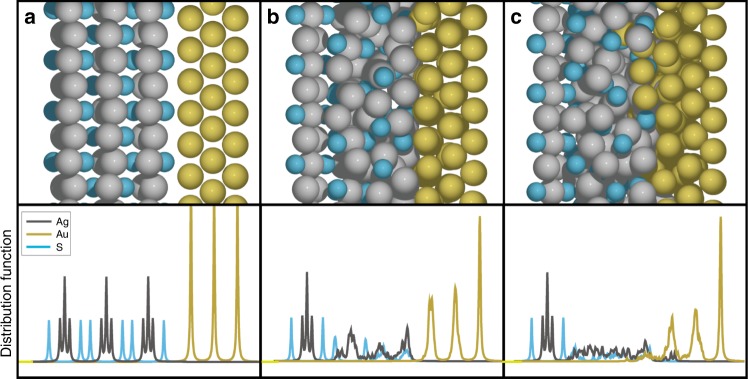
Molecular dynamics simulation of the welding process. Snapshots (top) and element distribution profiles (bottom) of inter-facial region of the nanostructures **a** before welding, **b** after 16 ps simulation of welding at 300 K, and **c** after 16 ps simulation of welding at 800 K. Color: Ag—silver; Au—gold; S—cyan

Ab initio molecular dynamics simulations were then employed to gain a deeper understanding of the welding mechanism. The two phases were modeled by a slab interface between (5 × 3) Ag_2_S(101) and (8 × 8) Au(111). Although no obvious welding was observed at reaction temperature (300 K) probably due to the limited length of simulation time (16 ps), one can still observe that the Ag_2_S phase becomes disordered (Fig. 3a, b). Previous studies have suggested that increase of simulation temperature can be used to accelerate the sampling of system with high energy barrier, thus enabling the modeling of slow reactions that are otherwise beyond the time scale of simulation^39^. We, therefore, used a simulation temperature of 800 K to observe the welding process. High-temperature simulation did confirm the welding phenomenon, with Au penetrating into the Ag_2_S matrix, followed by Ag filling into the empty sites of the Au slab (Fig. 3c). No obvious penetration of S into Au matrix was observed. Moreover, the simulation indicates the diffusion of Au into Ag_2_S matrix is easier than Ag into Au matrix (Supplementary Fig. 11), which might be attributed to the more flexible nature of Ag_2_S layered structure than the close packed fcc Au. Indeed, the barrier for Ag atom running out of Ag_2_S surface is only 0.80 eV, which is much lower than the barrier (1.45 eV) for Au atom moving out of Au(111). Combined with the EDS mapping results, we hypothesized that the diffusion of Au atoms into Ag_2_S lattice was the primary driving force for the welding process.

The coalescence between Au and Ag_2_S may occur via an Ostwald ripening process, which involves the dissolution of Au NPs and re-nucleation and growth on the surface of Ag_2_S NPs^40^. The ripening process is negligible in our case due to the relative large size of Au NPs (14 nm); however, it could be dominant for 8 nm Au NPs which are thermodynamically more unstable (Supplementary Fig. 12). This phenomenon was confirmed by the time-dependent analysis of metal ion concentration by inductively coupled plasma mass spectrometry: the dissolved Au ions significantly increased as a function of time for the 8-nm NP system, while the concentration of Au ions in the 14-nm system remained relatively low and constant throughout the welding process (Supplementary Table 2).

### Welding of NPs with different shapes and compositions

**Fig. 4:**
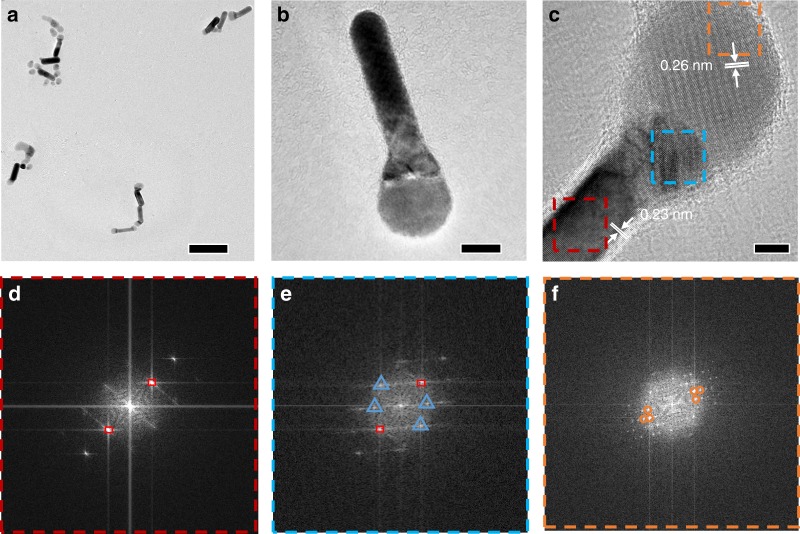
Formation of Au–Ag_2_S matchstick-like HNPs through welding of Au NRs and spherical Ag_2_S NPs. **a**–**c** TEM images of HNPs obtained by welding Au NRs and Ag_2_S NPs at different magnifications. **d**–**f** Corresponding FFT pattern of different regions in **c**: Au region in red box (**d**), welding region in blue box (**e**), and Ag_2_S region in orange box (**f**).The red squares, orange circles, and blue triangles in **d–f** highlighted the FFT patterns of Au, Ag_2_S, and a new phase, respectively. Scale bars are 100 nm in **a**, 10 nm in **b**, and 5 nm in **c**

The welding process was also observed in NPs with distinct shapes and compositions. When shaped NPs were used, the welding process led to the formation of structurally more complex HNPs. For instance, when the dispersion of Au NRs (40 nm in length and 10 nm in diameter) was mixed with the dispersion of Ag_2_S NPs, Ag_2_S NPs preferentially attached to the tips of Au NRs to form matchstick-like HNPs (Fig. 4a, b and Supplementary Fig. 13). We presume that the preferential welding of Ag_2_S at the ends of Au NRs is attributed to the deprivation of cetyltrimethylammonium bromide bilayer at the rod ends^41^. This is also supported by the fact that the Au NRs would homogeneously aggregate in an end-to-end manner in the absence of Ag_2_S NPs (Supplementary Fig. 14). HRTEM image showed lattice fringe of an individual HNP (Fig. 4c): the 0.23 nm interplanar spacing corresponded to Au (111) facet and the 0.26 nm spacing could be assigned to (−121) facet of monoclinic Ag_2_S. In between the Au and the Ag_2_S domain, an Au diffusion region as aforementioned could be clearly observed. The fast Fourier transform (FFT) patterns of this region (Fig. 4e, blue triangles) were distinct from those of Au (Fig. 4d, red squares) and Ag_2_S (Fig. 4f, orange circles), indicating the formation a new phase (presumably Ag_3_AuS_2_).

**Fig. 5:**
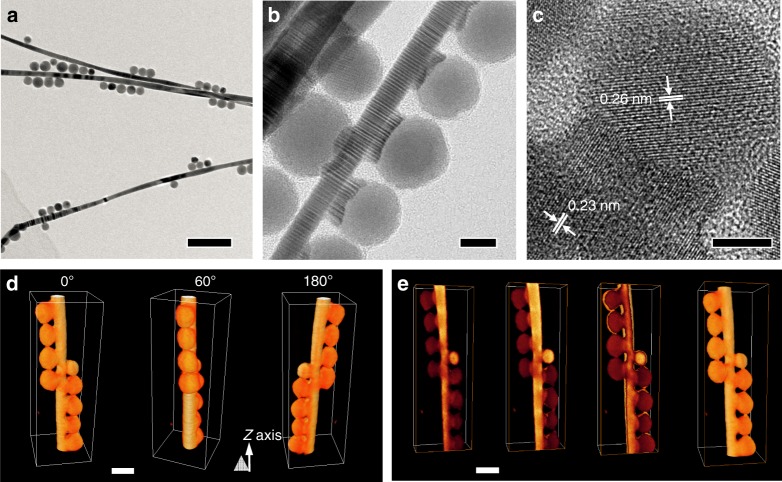
Formation of Au–Ag_2_S tree burl-like HNPs through welding of Au NWs and spherical Ag_2_S NPs. **a–c** TEM images of HNPs obtained by welding Ag_2_S NPs and Au NWs at different magnifications. **d** The volume renderings of the tomographic reconstruction of such HNPs at different rotation angles along *Z* axis. **e** Three-dimensional tomographic reconstruction of such Au–Ag_2_S HNPs. The progressing cross-sections and the isosurfaces visualize the internal structures of the nanocomposite. Scale bars are 100 nm in **a**, 10 nm in **b**, 5 nm in **c**, and 20 nm in **d** and **e**

When oleylamine (OAm) covered Au NWs (8 nm in diameter)^42^ and spherical Ag_2_S NPs were used, their welding upon desorption of OAm^19^ led to the formation of tree burl-like HNPs in which Ag_2_S NPs selectively attached to the sides of NWs (Fig. 5a, b). Similar to Au NPs and NRs, the diffusion of Au into Ag_2_S was also observed in HRTEM (Fig. 5c) and EDS mapping (Supplementary Fig. 15). The three-dimensional reconstruction of welded tree burl-like HNPs was obtained by using aberration-corrected scanning TEM (AC-STEM). TEM images of the nanostructure were taken every 2° when tilting from −70° to +70° (see Supplementary Movie 1) and used to reconstruct the 3D model (Fig. 5d). Figure 5e shows the isosurfaces at different cross-sections of the HNPs (see corresponding movie in Supplementary Movie 2), further indicating the welded rather than overlapped structures. Interestingly, the use of ultrathin Au NWs (2 nm in diameter) led to the simultaneous ripening and welding process (Supplementary Fig. 16).

**Fig. 6:**
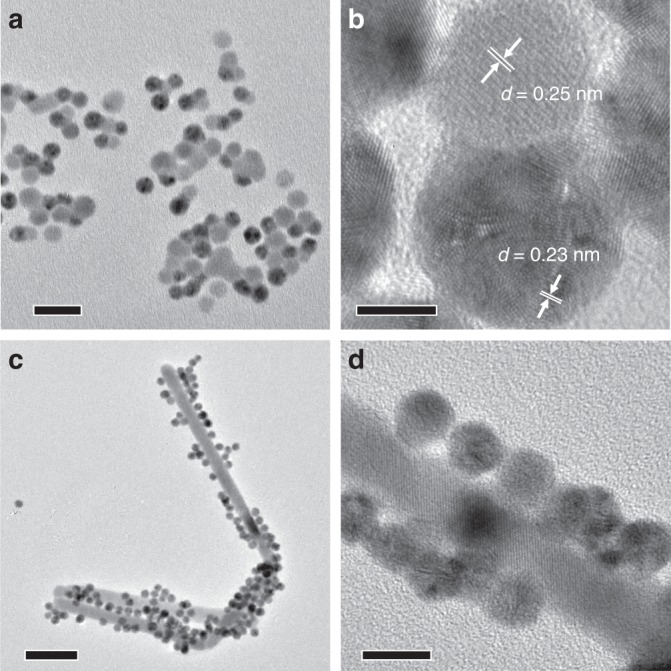
TEM images showing the welding between Au NPs and different chalcogenide NPs. **a**, **c** Low and **b**, **d** high magnification TEM images of **a**, **b** Au–Ag_2_Se HNPs welded from spherical Au and Ag_2_Se NPs and **c**, **d** Au–Ag_2_S HNPs welded from spherical Au and rod-like Ag_2_S NPs. Scale bars are 20 nm in **a**, 5 nm in **b**, 50 nm in **c**, and 10 nm in **d**

Selenides such as Ag_2_Se could also weld with Au NPs in dispersion to form HNPs (Fig. 6). When spherical Ag_2_Se capped with octadecylamine (ODA) was mixed with OAm-capped Au NPs in toluene, similar oligomer-like HNPs could be collected (Fig. 6a). The 0.23 and 0.25 nm d-spacing could be assigned to the (111) facet of Au and the (002) facet of Ag_2_Se, respectively (Fig. 6b). We also investigated the welding between spherical Au NPs and chalcogenides with different shapes. For instance, rod-like Ag_2_S NPs were prepared use a BSA-directed growth method with modification. Upon mixing with Au NPs in aqueous dispersion and subsequent incubation, the Au NPs preferentially attached to the sides of Ag_2_S NRs, forming similar tree burl-like HNPs as in the case of Au NW–Ag_2_S NPs (Fig. 6c). Notably, HRTEM image revealed the diffusion from Au NPs to Ag_2_S NRs (Fig. 6d).

## Discussion

In conclusion, we have revealed a unique welding phenomenon between Au and chalcogenides NPs in dispersion. The surface ligand desorption and Au diffusion are two major criteria that drive the welding process. The welding mechanism was investigated by a combination of tools, including EDS mapping, molecular dynamic simulation, and ESR measurements. We showed that the Au diffusion-dominated welding process can occur between NPs with distinct shapes or different capping agents or in different solvent media. In current experiments, the welding process produced HNPs with limited control over their nuclearity (i.e., with a defined number of NP precursors fused into a single entity) and the yield of hetero-fused structures (i.e., from distinct NPs) is not sufficiently high. Thus, before the welding process can be utilized for practical synthesis, future efforts should be made on identifying the criteria to improve the product yield and purity of welded HNPs. For instance, better engineering the interactions between NP precursors (e.g., electrostatic attraction between dissimilar NPs and repulsion between same-kind NPs) may lead to high selectivity in the association and fusion of NPs to produce uniform HNPs with controlled morphologies. Nevertheless, the welding process represents an important addition to the existing synthetic approach toward the fabrication of metal-chalcogenide HNPs with well-defined nanostructures, although future efforts are needed to further improve the yield of synthesis. These HNPs may find applications such as solar energy harvesting and conversion, photoluminescence, surface-enhanced Raman scattering, and biological and medical applications.

## Methods

### Synthesis of Au NPs

Typically, 10 mg of HAuCl_4_ was dissolved in 100 ml of H_2_O and heated to boiling under stirring. 3 ml of sodium citrate (1 wt%) solution was then quickly injected. The reaction mixture was refluxed for 30 min. Au NPs were collected by centrifuging the above solution at 9500 rpm for 15 min and re-dispersed in 20 ml of water.

### Preparation of PEG-Au NPs

For PEG surface modification of Au NPs with different grafting densities, typically, 5 ml of the abovementioned aqueous dispersion of Au NPs was added in four separate vials, followed by the addition of 10, 20, 40, and 80 µl of 1 mg ml^−1^ fresh prepared PEG-SH aqueous solution, respectively. The dispersions were stirred for 1 h, then incubated at ambient condition for 8 h. The dispersions were then directly used for NP welding. The PEG-Au NPs with different grafting densities were denoted as Au-1, Au-2, Au-3, and Au-4, respectively. The original Au NPs were concentrated to the same concentration with Au-1 and denoted as Au-0 for comparison. Detailed synthetic methods for Au NWs, Au NRs, Ag_2_S NPs, and Ag_2_Se NPs are shown in Supplementary Methods.

### Synthesis of BSA-Ag_2_S NPs and NRs

For Ag_2_S NPs, typically, 25 ml of 10 mM AgNO_3_ aqueous solution was mixed with 50 ml of 0.2 mg ml^−1^ BSA aqueous solution in a 250-ml concave flask in an ice bath. The mixture solution was stirred for 30 min and 25 ml of 10 mM thioacetamide (TAA) aqueous solution was subsequently added. The mixture solution was stirred in an ice bath for another 1 h before aging at room temperature for another 24 h. The solution was first centrifuged at 4000 rpm for 20 min, and the solids which were mainly Ag_2_S NRs were discarded. The supernatant was further centrifuged at 8000 rpm for 20 min, and Ag_2_S NPs were collected by re-dispersing the solids with 30 ml of water.

For Ag_2_S NRs, all conditions were same except that the growth solution was aged for 3 days. After that, the Ag_2_S NRs were collected by centrifuging the solution at 5000 rpm for 15 min.

Detailed synthetic methods for Au NWs, Au NRs, and Ag_2_Se NPs are shown in Supplementary Methods.

### Welding of Au and chalcogenide NPs

For the welding of Au NPs (or NRs) and Ag_2_S NPs, typically, 0.5 ml of aqueous dispersion of Au NPs (or NRs) was added into a 1.5-ml vial, followed by quick injection of 0.5 ml of aqueous dispersion of BSA-Ag_2_S NPs. The mixture dispersion was inversed vigorously and then incubated at room temperature for 24 h. For the welding of Au NWs and Ag_2_S NPs, typically, 0.1 ml of Au NWs in toluene was added into 0.8 ml of toluene in a 2-ml vial, followed by quick injection of 0.1 ml of Ag_2_S NPs in toluene. The mixture dispersion was capped and inversed vigorously, then incubated at room temperature for 48 h. For the welding of OAm-capped Au NPs and Ag_2_Se NPs, typically, 0.1 ml of Au NPs in toluene was added into 0.8 ml of toluene in a 2-ml vial, followed by quick injection of 0.1 ml of Ag_2_Se NPs in toluene. The mixture dispersion was capped and inversed vigorously, then incubated at room temperature for 48 h.

### Time-dependent ESR test of NP welding

The ESR measurements were carried out using a Bruker EMX ESR spectrometer (Billerica, MA) at ambient temperature. A solar simulator consisting of a 450 W Xenon lamp filtered to provide simulated sunlight was used in ESR studies. Fifty-microliter aliquots of control or sample dispersions were put into quartz capillary tubes with internal diameters of 0.9 mm and sealed. The capillary tubes were inserted in the ESR cavity, and the spectra were recorded before and after 15 min irradiation. All ESR measurements were carried out using the following settings for detection of the spin adducts: 20 mW microwave power, 100 G scan range, and 1 G field modulation. The sample solution consists of 45 µl of NP mixture dispersion and 5 µl of 10 uM TEMPO.

### Simulations

Ab initio molecular dynamics calculations were done with Vienna Ab-initio Simulation Package (VASP)^43,44^ at generalized-gradient approximation level with exchange-correlation functional in type of PBE^45^. The interaction between the atomic cores and electrons was described with the projector augmented wave (PAW) method^46^. The valence wave functions were expanded by plane-wave with a cutoff energy of 200 eV, within the software suggested ranges. A canonic ensemble was chosen for molecular dynamics with 1.5 fs time step. The Ag_2_S and Au interfaces were modeled by (5 × 3) Ag_2_S (101) and (8 × 8) Au (111) with only 1.2% unitcell mismatch. Both have a three-layered thickness, with the most outside layer fixed.

### Characterizations

UV–Vis extinction measurements were performed using PERKIN LAMBDA 40 UV–Vis system. The structure and morphology of all NPs was analyzed by Hitachi SU-70 Analytical field emission gun SEM (FEG-SEM) operated at 5 kV and JEOL 2100 TEM operated at 200 kV accelerating voltage. HAADF images and EDS mapping were performed on Titan Cubed Themis G2 300 (FEI) using scanning TEM mode under 60 kV accelerating voltage of the electron beam. All the SEM samples were prepared by dispensing a drop of the HNP dispersion on silicon wafers and drying at room temperature. All the TEM samples were prepared by depositing a drop of HNP dispersion onto 300 mesh carboncoated copper grids and allowing solvent evaporation at room temperature.

## Supplementary information

Supplementary Information

Description of Additional Supplementary Files

Supplementary Movie 1

Supplementary Movie 2

## Data Availability

The data that support the findings of this study are available from the corresponding authors upon reasonable request.
