# Supplementary material for: A welding phenomenon of dissimilar nanoparticles in dispersion
Source: Nat Commun. 2019 Jan 15;10:219. doi: 10.1038/s41467-018-08206-6 (PMC6333817; doi:10.1038/s41467-018-08206-6)
Supplement: Supplementary file 1 — Supplementary Information [file 41467_2018_8206_MOESM1_ESM.pdf]

Huang, et al. A welding phenomenon of dissimilar nanoparticles in dispersion

Supplementary Figures

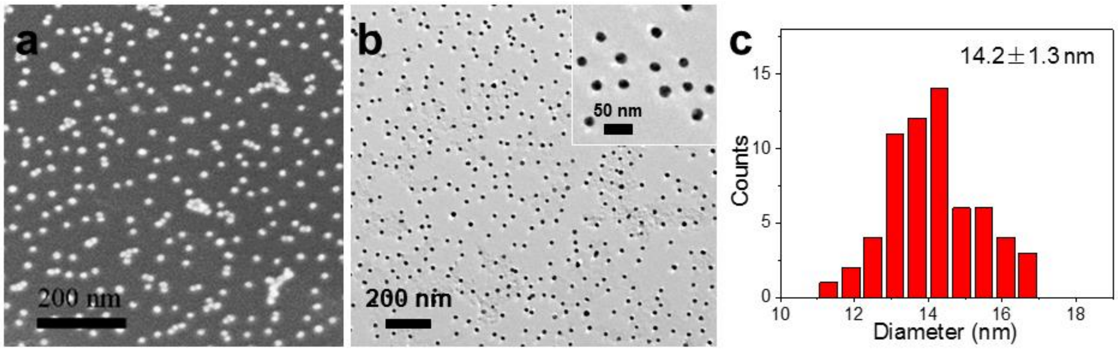

Supplementary Figure 1 | SEM, TEM images and size distribution of as-prepared Au NPs.

(a) SEM image, (b) TEM image and (c) histograms of diameter distribution by counting over 50 NPs.

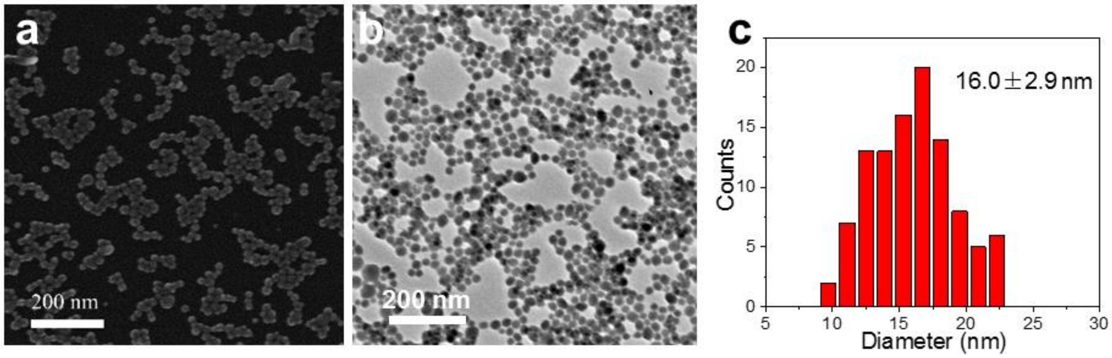

Supplementary Figure 2 | SEM, TEM images and size distribution of as-prepared Ag_2S NPs. (a) SEM image, (b) TEM image and (c) histograms of diameter distribution by counting over 50 NPs.

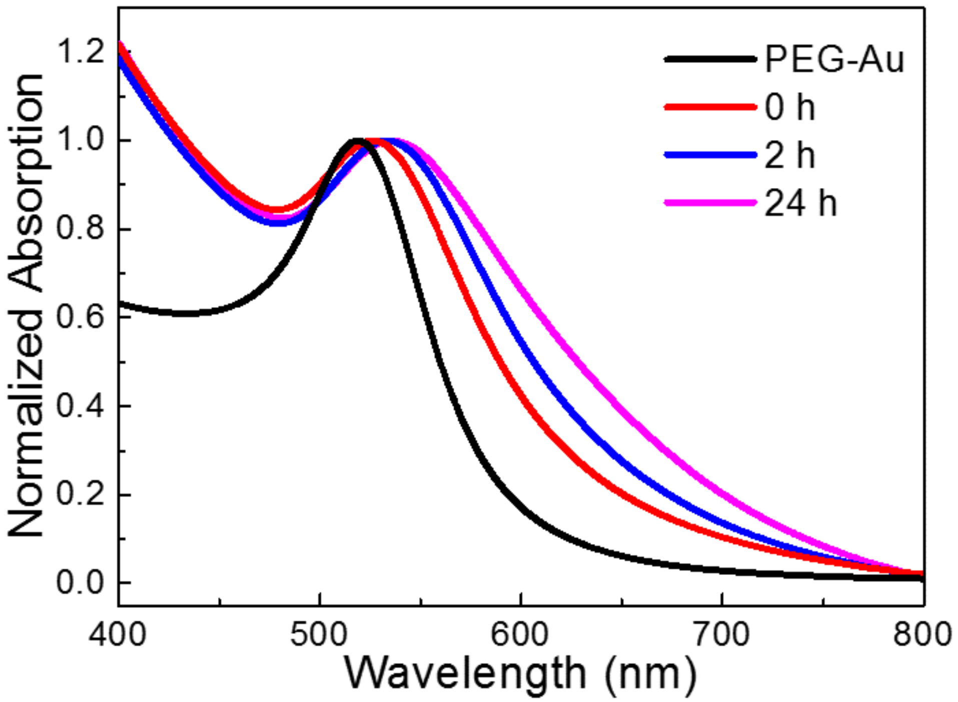

Supplementary Figure 3 | Normalized extinction spectrum of PEG grafted Au NPs and there mixture with Ag_2S NPs aged for different time. The surface plasmon resonance (SPR) peak of Au NPs red-shifted from 510 nm to 525 nm after welding, presumably due to the increase of local refractive index around Au NPs after Ag_2S NP attachment.

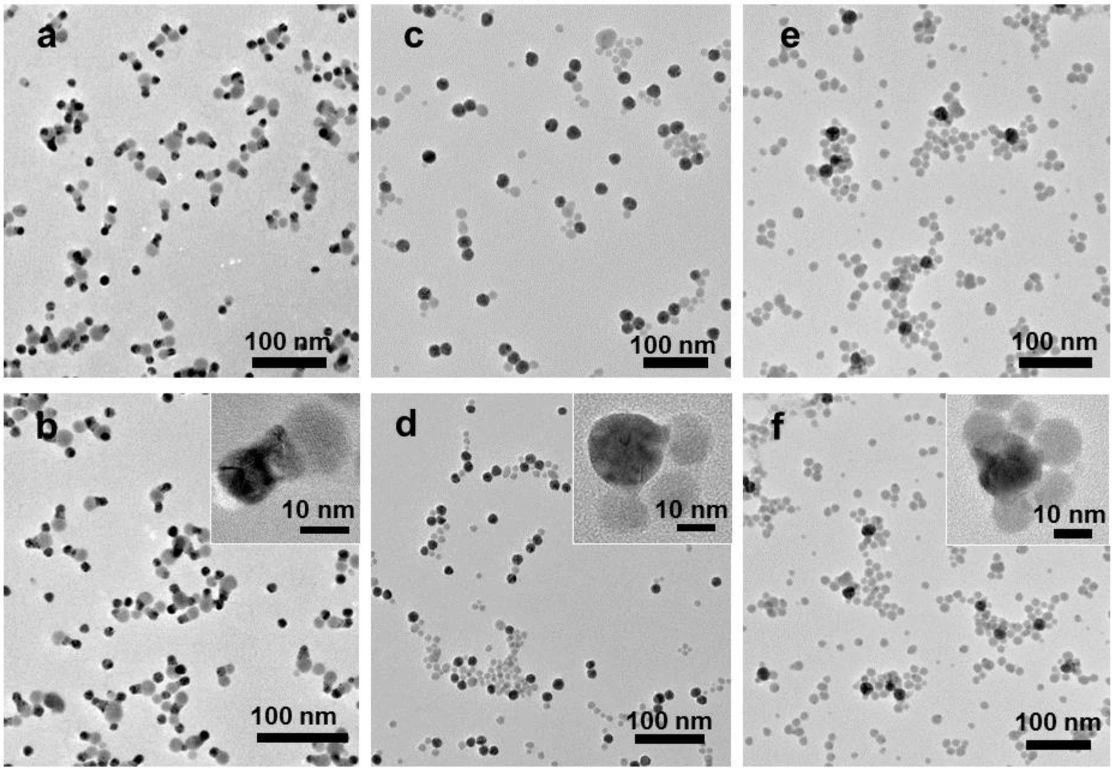

Supplementary Figure 4 | Representative TEM images of derived Au-Ag2S Oligomers with different Au: Ag2S feeding ratio. (a, b) 1: 1, (c, d) 1: 2 and (e, f) 1: 25. Insets are corresponding HRTEM images confirming the welding.

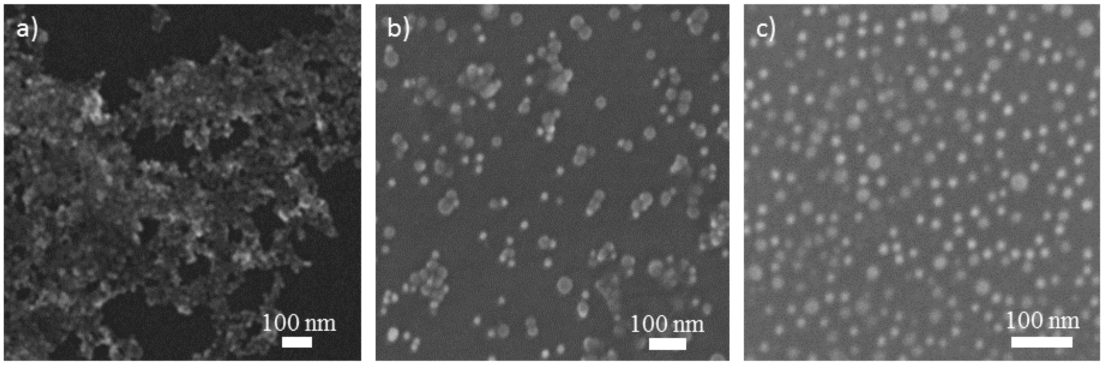

Supplementary Figure 5 | SEM images of Au-Ag_2S mixture solution with different PEG coverage on the surface of Au NPs. The PEG grafting density of Au NPs used for welding were (a) 0 (Au-0), (b) 0.11 (Au-2) and (c) 0.70 (Au-4), respectively

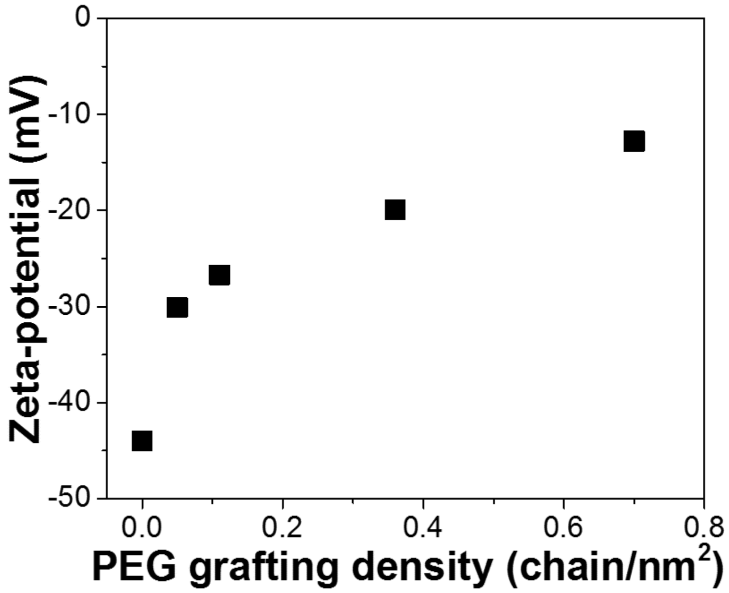

Supplementary Figure 6｜ Zeta-potential of Au NPs with different PEG grafting densities.

The surface charges of Au NPs decrease significantly with increasing PEG coverage (from Au-0 to Au-4).

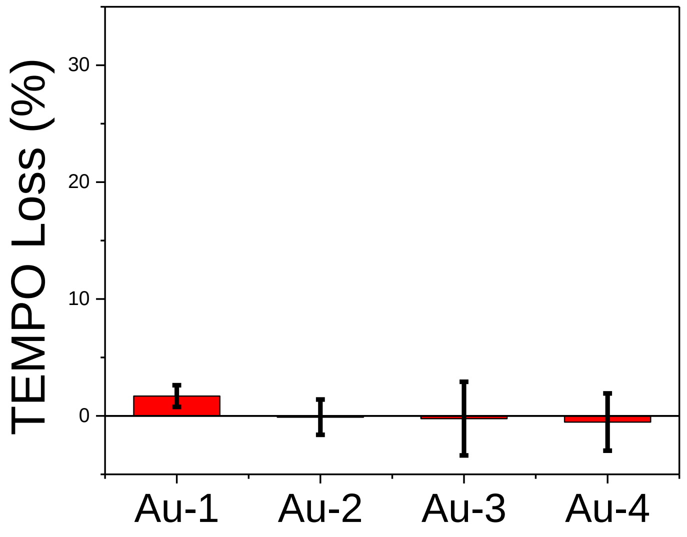

Supplementary Figure 7 | TEMPO Loss in the solution of Au NPs with different surface coverages of PEG, after the irradiation with visible light for 15 min. Each test is repeated 3 times. Standard deviations are represented by error bars.

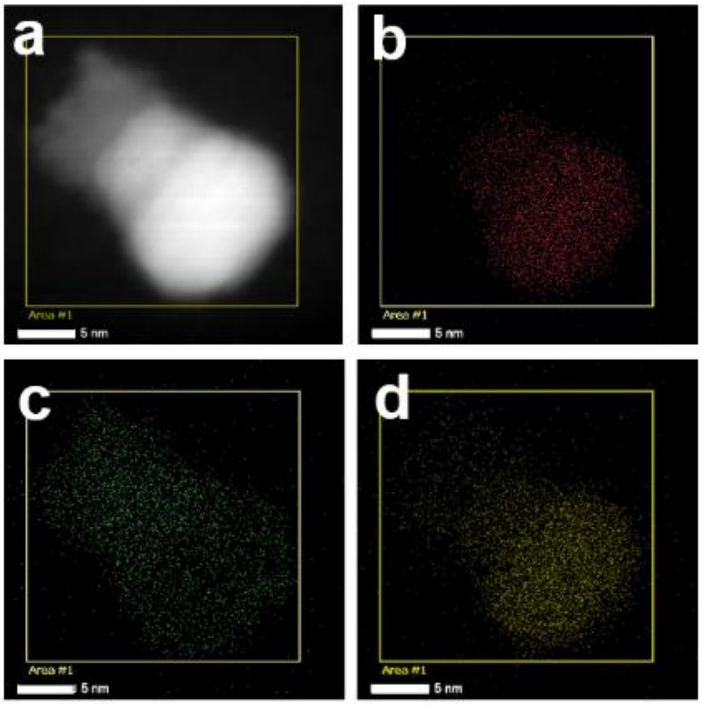

Supplementary Figure 8 | Structure characterization of welded Au-Ag_2S HNPs. HAADF image (**a**) and corresponding elemental distribution of Au (**b**), Ag (**c**), and S (**d**) in an individual Au-Ag_2S HNP. Scale bars are 5 nm.

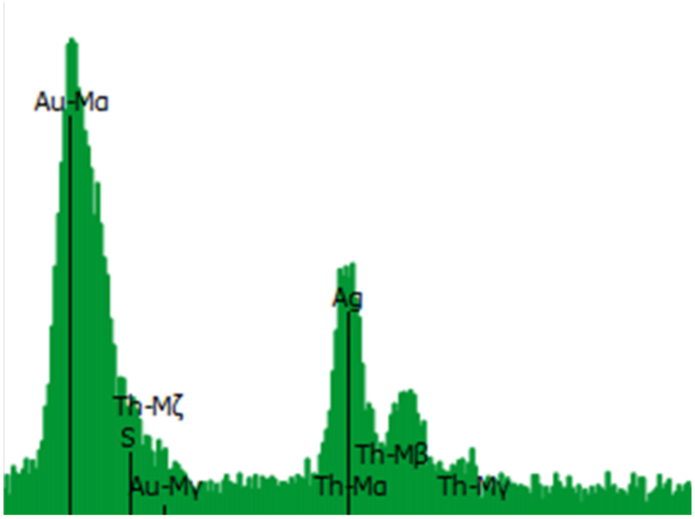

Supplementary Figure 9 | EDS spectrum of welded Au-Ag2S HNPs. The S peak is located on the shoulder of Au peak, thus the signal of S in the mapping largely comes from that of Au.

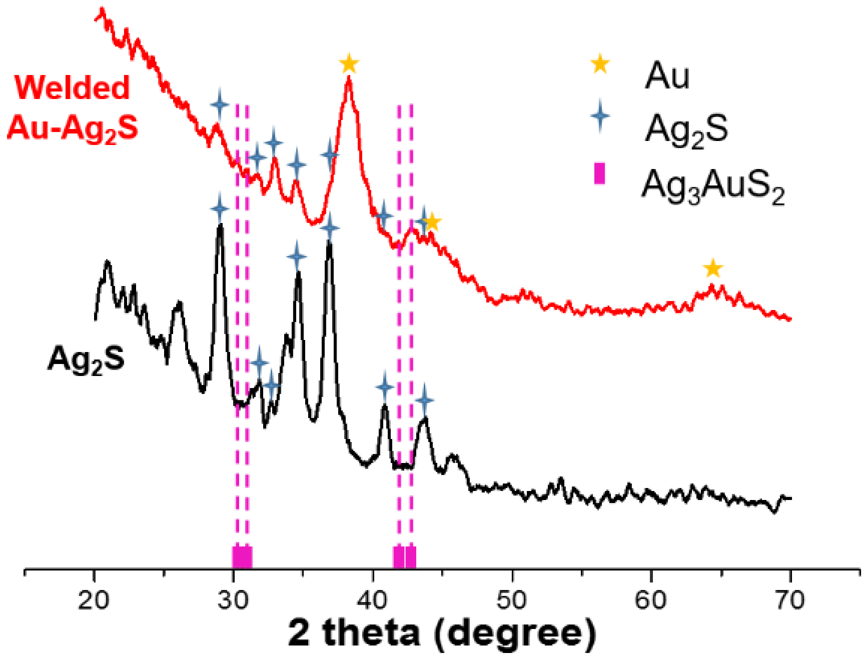

Supplementary Figure 10 | Structure characterization of welded Au-Ag_2S HNPs. XRD analysis of Ag_2S NPs and welded Au-Ag_2S HNPs. The peaks of Au, Ag_2S, and Ag_3AuS_2 are marked with yellow stars, blue stars, and purple dashed lines, respectively.

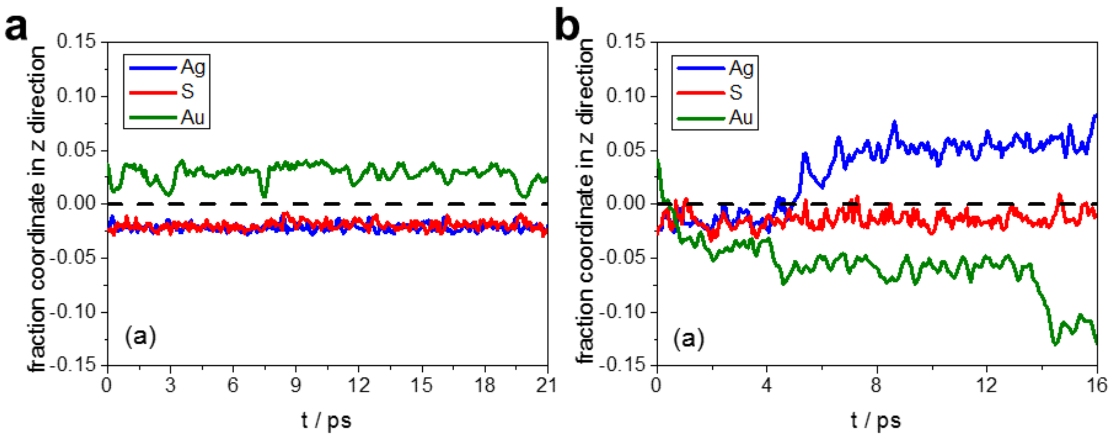

Supplementary Figure 11 | The location of the Ag, S and Au atoms which penetrate most into the other phases. (a) 300k, (b) 800k. Dashed line indicates the location of the Au/Ag_2S interface, with Au slab in the positive z quadrant ad Ag_2S in the negative z quadrant.

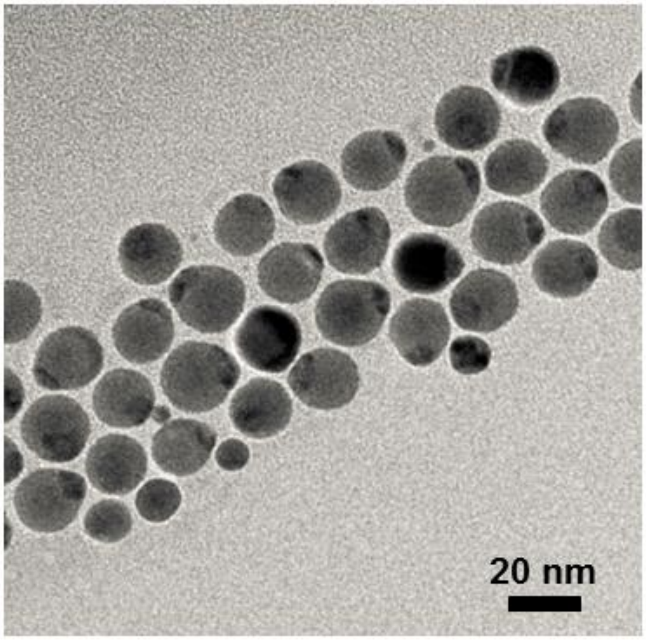

Supplementary Figure 12 | The Ostwald ripening between Ag_2S NPs and 8 nm Au NPs.

Small Au NPs tend to dissolve and re-nucleate and grow on the surface of Ag_2S NPs, forming small Au patches on Ag_2S NPs.

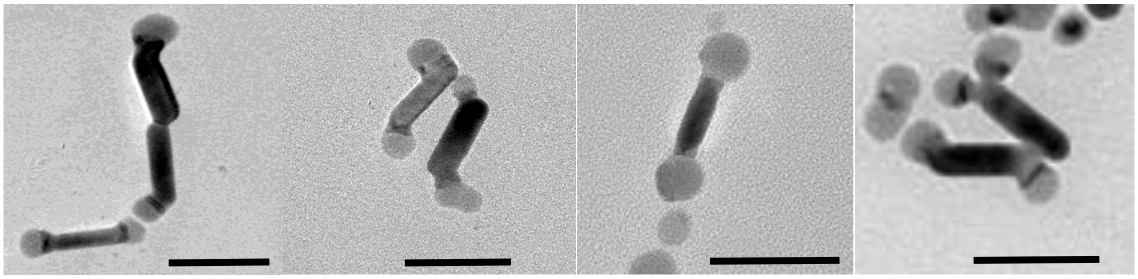

Supplementary Figure 13 | TEM images of matchstick-like Au-Ag_2S NPs formed via welding Au NRs and Ag_2S NPs. Scale bars, 50 nm.

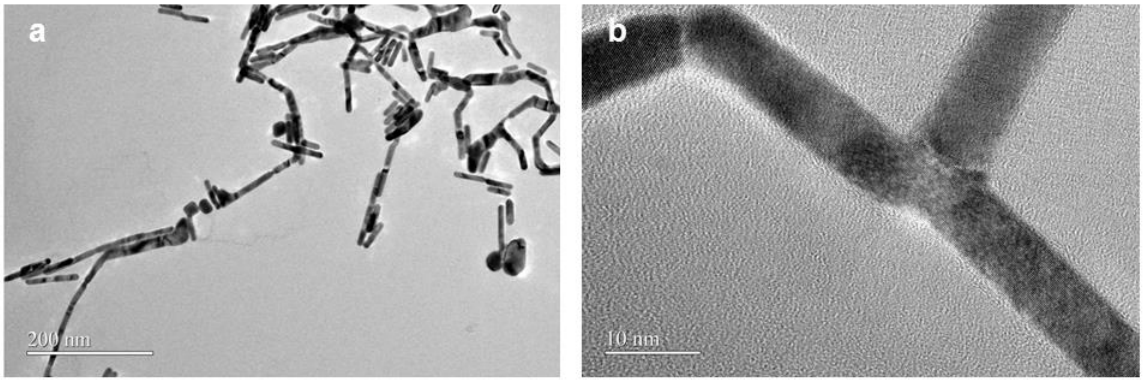

Supplementary Figure 14 | End-to-end welded Au NRs in absence of Ag_2S NP. (a) Large scale TEM image of end-to-end welded Au NRs, (b) HRTEM image of the joining part of three Au NRs.

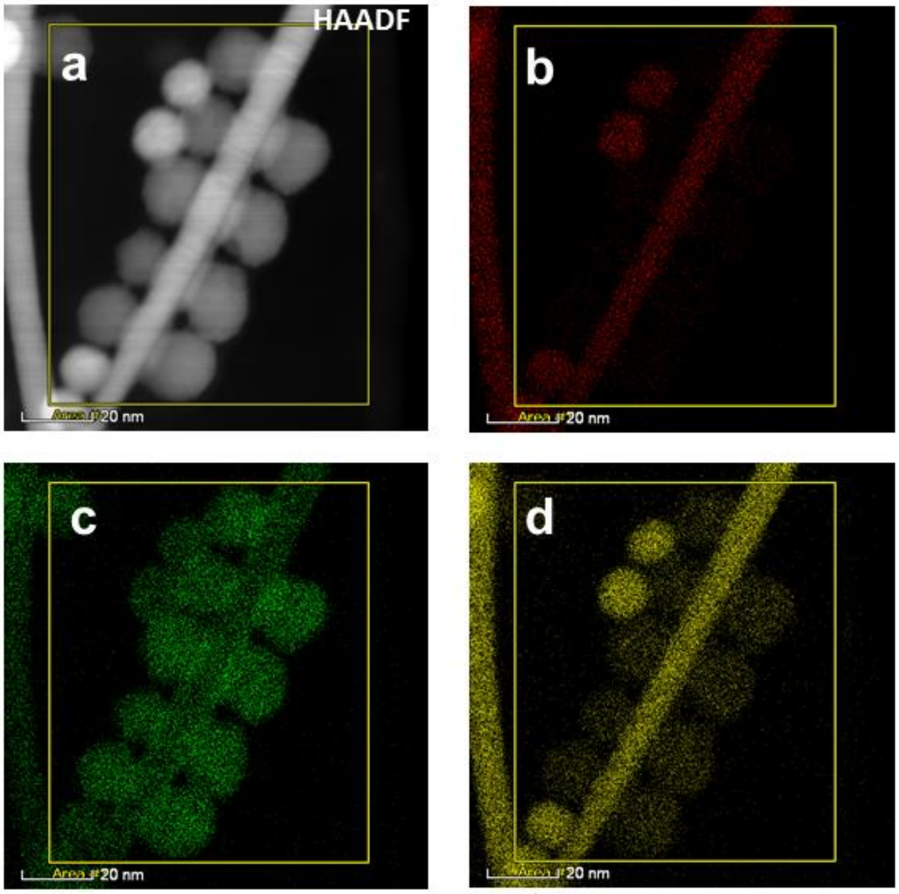

Supplementary Figure 15 | Elemental mapping of welded Au NW-Ag_2S HNP. (a) HAADF image and corresponding elemental distribution of (b) Au, (c) Ag, and (d) S in an individual Au NW-Ag_2S HNP.

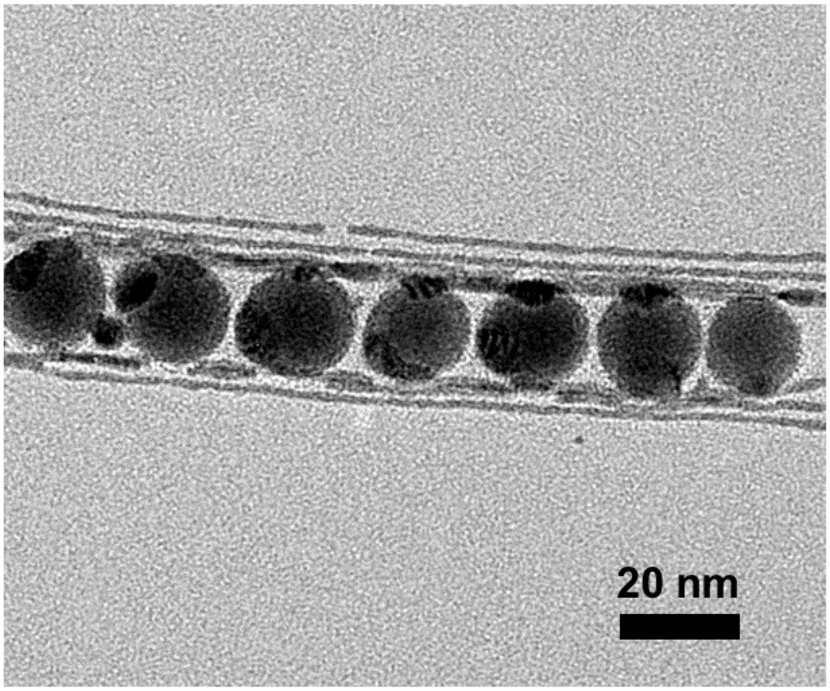

Supplementary Figure 16 | Simultaneous welding and ripening between ultrathin Au NWs (2 nm in diameter) and Ag_2S NPs.

Supplementary Tables

Sample	Mass of PEG (mg)	Mass of Au NPs (mg)	Grafting density (chain nm^{-2})
Au-1	0.008	0.752	0.05
Au-2	0.026	1.123	0.11
Au-3	0.051	0.717	0.36
Au-4	0.031	0.225	0.70

Supplementary Table 1｜ The mass of PEG and Au NPs measured from TGA and calculated polymer grafting density.

Incubation time	0 h	12 h	24 h
Au (mg L^{-1})-Sample 1	0.932	1.018	0.954
Ag-Sample 1	Undetectable	Undetectable	Undetectable
Au (mg L^{-1}) -Sample 2	0.880	N.A.	8.035
Ag-Sample 2	Undetectable	Undetectable	Undetectable

Supplementary Table 2 | The concentration of Au and Ag ions in solution at different incubation time points for the 14-nm Au NP system (Sample 1) and 8-nm Au NP system (Sample 2).

Supplementary Methods

Regents

Gold(III) chloride trihydate ($HAuCl_4$•$3H_2O$, 99.9+%), Silver nitrate ($AgNO_3$, 99+%), Thioacetamide (TAA, 99+%), Hexadecyltrimethylammonium bromide (CTAB, 99%+), Sodium borohydride ($NaBH_4$, 99.99%), L-ascorbic acid (AA, 99%+), sodium citrate dehydrate (SC, 99.0+%), were purchased from Sigma-Aldrich. 2,2,6,6-tetramethylpiperidine-1-oxyl (TEMPO) was purchased from Alexis. Toluene (99.5%+), hexane (99.0%+), oleic acid (OA, 99.0%+), sulfur (S, 99.0%+), selenium (Se, 99.0%+), octadecylamine (ODA, 99.0%+) and oleylamine (OAm, 99.5%+) were purchased from J&K. Thiol-terminated poly (ethylene glycol) (PEG-SH, M_n = 5000, M_w/M_n = 1.08) were purchased from Polymer Source Inc. All above chemicals were used as received without further processing. Deionized water (Millipore Milli-Q grade) with resistivity of 18.0 MΩ was used in all the experiments.

Sample preparation for Thermal gravimetric analysis (TGA)

PEG modified Au NPs were centrifuged with water twice to remove free PEG. The derived solids were dried at 100 °C oven for 12 hours to remove the solvent residues before TGA measurement.

Polymer grafting density calculation

The mass of Au and PEG was measured by TGA. The number of Au NPs in the sample can be calculated by Supplementary Equation 1:

$$N_{NP} = \frac{mass\ of\ Au}{\rho_{Au} * V_{NP}} \qquad (1).$$

where ρ_{Au} is the density of gold and V_{NP} is the volume of a single Au NP.

The number of polymer chains on the surfaces of Au NPs can be calculated by Supplementary Equation 2:

$$N_{PEG} = \frac{mass\ of\ PEG}{M_w\ of\ PEG} * N_A \qquad (2).$$

where N_A is the Avogadro's number.

The polymer grafting density can be calculated by Supplementary Equation 3:

$$\delta = \frac{N_{PEG}}{N_{NP} * A_{NP}} \qquad (3).$$

where A_{NP} represents the surface area of a single Au NP.

Synthesis CTAB capped Au nanorods

CTAB capped Au nanorods were synthesized with a modified seed-mediated growth method[1]. A seed solution was first prepared as follows: a 2.5 ml of 0.2 M CTAB aqueous solution and a 0.125 ml of 10 mM $HAuCl_4$ aqueous solution were sequentially added into 2.375 ml of water in a 20 ml scintillation vial, followed by injection of a 0.3 ml of ice-cold 10 mM $NaBH_4$ aqueous solution in one shot under vigorous stirring. The solution immediately turned dark brown. After stirring for 2 minutes at room temperature, the solution was held in a 29 °C water bath for 2 hours before use.

The growth solution was prepared in a 250 ml conical flask. A 95 ml of 0.1 M CTAB aqueous solution, a 1 ml of 10 mM $AgNO_3$ aqueous solution, and a 5 ml of 10 mM $HAuCl_4$ aqueous solution were sequentially added to the flask, followed by injecting a 0.6 ml of 0.1 M AA aqueous solution. Upon AA injection, the flask was gently shaken till the solution turned clear. Finally, a 1.6 ml of Au seed solution was added. After gently shaking, the flask was left in a 29 °C water bath overnight.

Synthesis of Au NWs

Au NWs with different diameters were synthesized according to previous report[2]. For Au NWs with 8 nm diameter, typically, a solution (containing 1 ml hexane and 1 ml OAm) of 0.1 g of $HAuCl_4$ was added to the mixture of OA (5 mL) and OAm (4 mL) at 80 °C under vigorous magnetic stirring. Hexane was evaporated under nitrogen atmosphere. Magnetic stirring was stopped after 5 min, and the solution was kept steady at this temperature for 5 h. The Au NWs were precipitated out by adding ethanol and centrifugation. The dark solids were washed twice with ethanol and redispersed in 10 ml of toluene. For ultrathin Au NWs with 2 nm diameter, everything was same as the method described above, except for the absence of OA.

Synthesis of OAm-Ag_2S NPs

Typically, 50 mg of silver nitrate was dissolved in 2 ml of oleylamine solution under argon at room temperature. This solution was injected to sulfur (50 mg) solution of 7 ml oleylamine at 100°C. The color of the solution rapidly became black. The reaction mixture was stirred for 5 hours and cooled to room temperature. The NPs were precipitated and washed with ethanol, and redispersed in toluene.

Synthesis of ODA-Ag_2Se NPs

ODA capped Ag_2Se NPs were prepared according to previous report3. Briefly, 0.5 g of $AgNO_3$ was added to 10 ml of ODA at 180 °C. The mixture was magnetically stirred for 10 min in air before 0.12 g of Se was added. The mixture solution was stirred for another 10 min. Ag_2Se NPs were collected and washed several times with ethanol and then dispersed in toluene.

Synthesis of OAm-Au NPs

OAm-Au NPs were prepared according to previous report[3]. Typically, a solution of 50 mg of $HAuCl_4$•$3H_2O$ in 1.2 ml of OAm and 1.0 ml of toluene into a boiling solution 2.9 ml of OAm in 49 ml of toluene. Heating was stopped after 2 hours, and 100 mL of methanol were added to precipitate the product. The particles were isolated by centrifugation, and washed three times with 50 ml portions of methanol and redispersed in 10 ml of toluene.

Supplementary References

1. Sau, T.K. & Murphy, C.J. Seeded High Yield Synthesis of Short Au Nanorods in Aqueous Solution. *Langmuir* **20**, 6414-6420 (2004).
2. Wang, C., Hu, Y., Lieber, C.M. & Sun, S. Ultrathin Au Nanowires and Their Transport Properties. *J. Am. Chem. Soc.* **130**, 8902-8903 (2008).
3. Hiramatsu, H. & Osterloh, F.E. A Simple Large-Scale Synthesis of Nearly Monodisperse Gold and Silver Nanoparticles with Adjustable Sizes and with Exchangeable Surfactants. *Chem. Mater.* **16**, 2509-2511 (2004).
