# Supplementary material for: A welding phenomenon of dissimilar nanoparticles in dispersion
Source: Nat Commun. 2019 Jan 15;10:219. doi: 10.1038/s41467-018-08206-6 (PMC6333817; doi:10.1038/s41467-018-08206-6)
Supplement: Supplementary file 2 — Description of Additional Supplementary Files [file 41467_2018_8206_MOESM2_ESM.pdf]

Description of Additional Supplementary Files

File Name: Supplementary Movie 1
Description: A 360-degree rotation movie along axial direction of the nanowire showing the three dimensional view of a welded Au-Ag_2S nanowire.

File Name: Supplementary Movie 2
Description: Three-dimensional tomographic reconstruction of a welded Au-Ag_2S nanowire with progressing cross sections.
